# Modifications of peripheral perfusion in patients with vasopressor-dependent septic shock treated with polymyxin B-direct hemoperfusion

**DOI:** 10.1038/s41598-023-34084-0

**Published:** 2023-05-05

**Authors:** Motohiro Sekino, Yu Murakami, Shuntaro Sato, Ryosuke Shintani, Shohei Kaneko, Naoya Iwasaki, Hiroshi Araki, Taiga Ichinomiya, Ushio Higashijima, Tetsuya Hara

**Affiliations:** 1 Department of Anesthesiology and Intensive Care Medicine, Nagasaki University Graduate School of Biomedical Sciences, 1-7-1 Sakamoto, Nagasaki, 852-8501 Japan; 2 Department of Anesthesiology, Nagasaki Harbor Medical Center, 6-39 Shinchi, Nagasaki, 850-8555 Japan; 3 Clinical Research Center, Nagasaki University Hospital, 1-7-1 Sakamoto, Nagasaki, 852-8501 Japan

**Keywords:** Sepsis, Continuous renal replacement therapy

## Abstract

Abnormal peripheral perfusion (PP) worsens the prognosis of patients with septic shock. Polymyxin B-direct hemoperfusion (PMX-DHP) increases blood pressure and reduces vasopressor doses. However, the modification of PP following administration of PMX-DHP in patients with vasopressor-dependent septic shock have not yet been elucidated. A retrospective exploratory observational study was conducted in patients with septic shock treated with PMX-DHP. Pulse-amplitude index (PAI), vasoactive inotropic score (VIS), and cumulative fluid balance data were extracted at PMX-DHP initiation (T0) and after 24 (T24) and 48 (T48) h. Changes in these data were analyzed in all patients and two subgroups (abnormal PP [PAI < 1] and normal PP [PAI ≥ 1]) based on the PAI at PMX-DHP initiation. Overall, 122 patients (abnormal PP group, n = 67; normal PP group, n = 55) were evaluated. Overall and in the abnormal PP group, PAI increased significantly at T24 and T48 compared with that at T0, with a significant decrease in VIS. Cumulative 24-h fluid balance after PMX-DHP initiation was significantly higher in the abnormal PP group. PMX-DHP may be an effective intervention to improve PP in patients with abnormal PP; however, caution should be exercised as fluid requirements may differ from that of patients with normal PP.

## Introduction

Initial resuscitation, consisting of the administration of fluids and noradrenaline, is a mainstay of current sepsis treatment guidelines to maintain organ perfusion and improve outcomes in patients with septic shock^1,2^. Lactate levels have been used as an indicator of tissue hypoperfusion, and the guidelines suggest reducing the lactate level as the target of resuscitation^1,2^. However, this parameter is not well established in resuscitation as elevated lactate levels are caused not only by tissue hypoperfusion but also by increased glycolysis associated with the activation of the stress response^3^.

In patients with septic shock, abnormal peripheral perfusion (PP), or peripheral hypoperfusion, has been associated with a particularly poor prognosis^4–7^. A recent study found that initial resuscitation aimed at improving abnormal PP as assessed by capillary refill time (CRT) resulted in a better prognosis than that aimed at improving lactate levels^8,9^. Therefore, the latest Surviving Sepsis Campaign Guidelines (SSCG) 2021 suggest using CRT to guide resuscitation as an adjunct to other perfusion measures such as lactate levels^2^. Interventions to improve abnormal PP have taken the same approach as those to improve lactate levels: administering dobutamine and milrinone in addition to fluid loading and increasing the noradrenaline dose to target high mean arterial pressure (MAP)^8^; however, higher MAP target does not improve PP^7^. Additionally, dobutamine is also ineffective in improving PP^10^, and although milrinone may improve PP, it carries the risk of lowering blood pressure^11,12^. Currently, no specific intervention has been established to improve abnormal PP in patients with septic shock.

Polymyxin B-direct hemoperfusion (PMX-DHP) is expected to improve pathophysiological derangements and reduce the mortality of patients with septic shock by adsorbing endotoxins, anandamide, and inflammatory cells^13–15^. However, its effectiveness in reducing mortality is unproven^16^, and its use is not recommended in the guidelines^1,2^. Meanwhile, several randomized controlled trials (RCTs) and meta-analyses have confirmed that PMX-DHP indeed increases blood pressure^16^, resulting in reduced doses of vasopressors^15^, which exert adverse effects on PP^17,18^. PMX-DHP reportedly induces vasoconstriction in patients with excessively dilated peripheral blood vessels by adsorbing anandamide, 2-arachidonylglycerol, and nitric oxide, thus contributing to improved hemodynamics^15,19^. However, modifications in PP following the initiation of PMX-DHP in patients with vasopressor-dependent septic shock, especially those with abnormal PP, have not yet been elucidated. In addition, PP modifications can alter blood flow distribution, which in turn may influence fluid balance^20^. Clarifying these questions may lead to effective use of PMX-DHP to reduce mortality.

Consequently, this retrospective exploratory observational study was conducted with the primary objective of investigating the modifications in PP after the initiation of PMX-DHP in patients with vasopressor-dependent septic shock. The secondary objective was to assess the vasopressor dosage and cumulative fluid balance after the initiation of PMX-DHP.

## Methods

### Study design

This single-center exploratory retrospective study was conducted at an eight-bed general intensive care unit (ICU) at the Nagasaki University Hospital (Nagasaki, Japan). This study was approved by the Nagasaki University Hospital Clinical Research Ethics Committee (No. 210913) and was conducted according to the Declaration of Helsinki. The requirement for written informed consent was waived by the Nagasaki University Hospital Clinical Research Ethics Committee owing to the retrospective study design.

### Study population

This study included adult (≥ 18 years of age) patients with vasopressor-dependent septic shock treated with PMX-DHP in an ICU of a tertiary care university hospital between January 2017 and December 2020. Septic shock was diagnosed according to the third International Consensus Definitions for Sepsis and Septic Shock criteria^21^. We excluded patients treated with PMX-DHP after 24 h of ICU admission, those who died or were discharged from the ICU after recovery within 48 h of ICU admission, and those with a terminal comorbidity, purpura fulminans, or missing data on PP.

### Clinical management

All patients were treated not only according to the SSCG2016^22^ and J-SSCG2016 Japanese guidelines^23^ but also with treatments, including PMX-DHP, that were not recommended in these guidelines. During the ICU stay, the entire treatment plan was left to the discretion of experienced intensivists. Fluid management was based on dynamic parameters such as arterial pulse pressure variation, response to infusion and passive leg raising, and variations in the diameter of the inferior vena cava^24^. Central venous pressure (CVP) was also measured to assess preload trends. Noradrenaline was used as a first-line vasopressor, with a target MAP of 65 mmHg or higher (80–85 mmHg MAP for patients with a medical history of hypertension). When noradrenaline administration was insufficient, vasopressin was administered. Low-dose hydrocortisone was also administered, as required. Cardiac function was evaluated, as appropriate, using echocardiography; if cardiac dysfunction was present or suspected, olprinone, which is a phosphodiesterase 3 inhibitor, dobutamine, or dopamine alone or in combination was used.

Other therapeutic interventions included landiolol, an ultrashort acting β1-receptor blocker used for tachyarrhythmia^25^, and low-dose carperitide, a human atrial natriuretic peptide, for vasodilation and diuresis, used as needed after circulatory stabilization^26^. Lung-protective ventilator support was performed as part of the management strategy. Antithrombin and recombinant soluble thrombomodulin were administered for septic disseminated intravascular coagulation (DIC)^1^. One or more antibiotics were administered as initial empirical treatment, which were then modified according to the culture results and clinical response.

### PMX-DHP treatment protocol

PMX-DHP was initiated principally when vasopressin and low-dose hydrocortisone were required in addition to noradrenaline doses of more than 0.3 μg/kg/min, despite the abovementioned interventions. Endotoxin levels were not used as an induction criterion for PMX-DHP because it is unmeasurable at our institution. Direct hemoperfusion with TORAYMYXIN PMX-20R (Toray Industries, Tokyo, Japan) was conducted at a 150 mL/min flow rate through a double-lumen central catheter. Nafamostat mesylate (Sawai Pharmaceutical Co., Ltd., Osaka, Japan), a protease inhibitor, was continuously administered as an anticoagulant agent at 20–40 mg/h to maintain the activated clotting time at approximately 150 s. PMX-DHP was usually used for 2 h per cartridge, but we used one cartridge for up to a maximum of 24 h as prolonged use reportedly contributes to a further improvement in hemodynamics in patients with septic shock^27–29^. In principle, two sessions (two cartridges) of PMX-DHP were administered. However, PMX-DHP could also be discontinued after the first session if the attending intensivist determined that there had already been an adequate improvement in hemodynamics. For patients with renal failure, continuous renal replacement therapy was administered simultaneously with PMX-DHP.

### Assessment of PP

PP was evaluated using the pulse-amplitude index (PAI) as measured with a pulse oximeter on a bedside monitor (Life Scope G9 or Life Scope J, NIHON KOHDEN Corp., Tokyo, Japan). At our institution, PAI has been measured and recorded for all patients and used as an indicator of PP since January 2017. The pulse oximeter probe was attached to the fingertips. PAI is calculated as the ratio of the pulsatile component over the non-pulsatile component of the transmitted infrared light intensity, using the same principle as the perfusion index^30^. The perfusion index is associated with changes in PP: values < 1.4 indicate hypoperfusion, while a value < 0.6 is an independent factor for mortality among critically ill patients, although neither has been firmly established^30–32^. Therefore, considering a previous report, we adopted a pragmatic threshold < 1.0 for abnormal PP in this study^33^.

### Endpoints and data collection

The primary endpoint was PAI within the first 48 h after PMX-DHP initiation in patients with vasopressor-dependent septic shock. The secondary endpoints were the vasoactive inotropic score (VIS)^34^ and 24- and 48-h fluid balance after PMX-DHP initiation. VIS was calculated as 100 × noradrenalin dose [µg/kg/min] + 10,000 × vasopressin dose [units/kg/min] + dopamine dose [µg/kg/min] + dobutamine dose [µg/kg/min] + 100 × adrenaline dose [µg/kg/min] + 25 × olprinone dose [µg/kg/min]^34^. The analysis was performed in all patients and in two subgroups according to the PAI at PMX-DHP initiation (abnormal PP [PAI < 1] and normal PP [PAI ≥ 1]). We also examined the correlation between PAI and VIS at initiation of PMX-DHP in all patients. Finally, we investigated whether PAI at PMX-DHP initiation was associated with subsequent 24- and 48-h cumulative fluid balance.

PAI values at three timepoints (at PMX-DHP initiation [T0] and after 24 [T24] and 48 [T48] h) were extracted from the ICU information system (Prescient ICU; FUJIFILM Medical Co., Ltd., Tokyo, Japan) along with VIS data (with noradrenaline and vasopressin dosages), MAP, heart rate, CVP, body temperature (axillary temperature), lactate level, and PaO_2_/F_I_O_2_ ratio. The 24- and 48-h cumulative fluid balance data after PMX-DHP initiation were also collected (including blood products, nutritional supplements, ultrafiltration volume, gastrointestinal loss, and drainage volume).

The baseline parameters on admission and within the first 24 h after admission to the ICU were also retrieved from the ICU information system and the electronic medical records system (MegaOakHR; NEC Corp., Tokyo, Japan). Data included information on age, sex, body mass index, Acute Physiology and Chronic Health Evaluation (APACHE) II score, Sequential Organ Failure Assessment score, and the Japanese Association for Acute Medicine DIC score^35^. Interventions for septic shock included the use of mechanical ventilation, continuous renal replacement therapy, low-dose hydrocortisone, soluble recombinant thrombomodulin, and antithrombin. Additionally, blood chemistry findings, including arterial levels of serum lactate and procalcitonin, were extracted on admission to the ICU to assess the severity of sepsis. Admission route to the ICU, surgical interventions before admission to the ICU, comorbidities, site of infection, causative microorganism, bacteremia status, all-cause 28-day mortality, and in-hospital mortality were also recorded.

Data on the time of initiation of PMX-DHP after admission to the ICU, number of sessions, and duration of treatment were also extracted from the ICU information system.

### Statistical analysis

Variables are presented as median and interquartile range for quantitative variables and as counts and percentages for categorical variables. The Wilcoxon rank-sum and Fisher’s exact tests were used to compare the two groups. PAI, VIS, MAP, vasopressor dose, heart rate, CVP, body temperature, lactate, and the PaO_2_/F_I_O_2_ ratio within the same group were compared using the Wilcoxon signed-rank test. The correlation between PAI and VIS at the time of PMX-DHP initiation was evaluated for all patients using Spearman’s correlation coefficient (ρ). Simple and multivariable linear regression analyses were performed to evaluate the association between the 24-h cumulative fluid balance per body weight and PAI at T0, age, APACHE II score, immunosuppression status, abdominal sepsis, and VIS at T0. Age, APACHE II score, immunosuppression status, and abdominal sepsis were included in the analysis because it has been suggested that these factors are related to the cumulative fluid balance in the first 24 h after admission to the ICU^36^. VIS at T0 was also included in the model from clinical relevance. All tests were two-sided, and *p*-values < 0.05 indicated statistical significance. All statistical analyses were performed using JMP Pro v16 (SAS Institute Inc., Cary, NC, USA).

### Ethics approval and consent to participate

Ethics approval of this retrospective study was provided by the Nagasaki University Hospital Clinical Research Ethics Committee (No. 210913), and the study was performed following the Declaration of Helsinki. The requirement for written informed consent was waived by the Nagasaki University Hospital Clinical Research Ethics Committee owing to the retrospective study design.

## Results

### Patient characteristics and implementation of PMX-DHP

**Figure 1:**
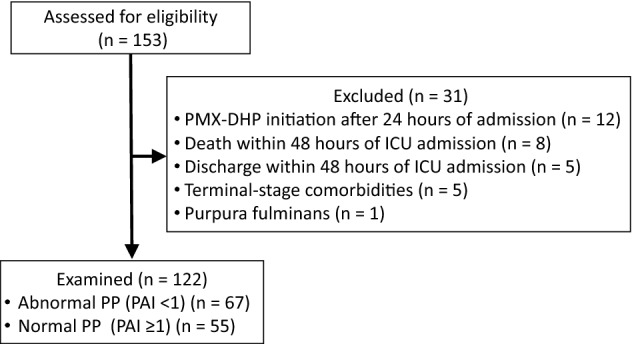
Flow diagram of included patients.

Overall, 153 patients with septic shock were considered for inclusion in this exploratory retrospective study; 122 were examined, and 67 and 55 were assigned to the abnormal and normal PP groups, respectively (Fig. 1). The characteristics of the patients are listed in Table 1 and Additional file 1. Most patients were older adults, severely ill, and required high-dose vasopressor administration, which was more pronounced in the abnormal PP group than in the normal PP group (Table 1). Since CVP values were missing for eight patients (four each in the normal and abnormal PP groups) at all measuring points during the study period, they were excluded from the analysis of CVP. There were no missing data for any other extracted parameters. There were no significant differences in other background factors between the two groups, including comorbidities, sources of infection, and causative organisms (Additional file 1).

**Table 1:** Baseline characteristics and clinical outcomes of the patients.

	All patients (n = 122)	Abnormal PP (PAI < 1) (n = 67)	Normal PP (PAI ≥ 1) (n = 55)	*p*-value
Age (years)	71 (64–79)	73 (67–80)	68 (63–77)	0.031
Sex, male, n (%)	69 (57)	35 (52)	34 (62)	0.359
Body mass index (kg/m^2^)	21.0 (19.0–24.7)	21.0 (19.1–25.0)	20.8 (18.5–24.4)	0.835
APACHE II score	28 (21–31)	28 (22–33)	25 (20–29)	0.029
SOFA score	12 (8–14)	13 (10–15)	11 (7–14)	0.074
Previous immunosuppression, n (%)	19 (16)	10 (15)	9 (16)	1.000
Site of infection				
Abdomen, n (%)	71 (58)	41 (61)	30 (55)	0.468
Lung/thorax, n (%)	22 (18)	10 (15)	12 (22)	0.352
Other/unknown, n (%)	29 (24)	16 (24)	13 (24)	1.000
JAAM DIC score	5 (3–6)	5 (3–6)	4 (2–6)	0.104
DIC, n (%)	68 (56)	39 (58)	29 (53)	0.586
Maximum lactate (mmol/L)	3.7 (2.6–5.8)	4.5 (3.0–6.3)	3.1 (2.2–4.8)	0.002
White blood cell (10^3^/µL)	7.5 (2.2–13.8)	7.6 (2.1–13.3)	6.9 (2.2–16.5)	0.907
Procalcitonin (mg/dL)	17.4 (5.0–66)	27.6 (5.4–71.3)	12.7 (4.4–41.0)	0.174
Baseline data at PMX-DHP initiation				
PAI (%)	0.78 (0.41–1.65)	0.42 (0.22–0.56)	1.71 (1.25–2.38)	< 0.0001
VIS	54 (38–66)	56 (46–71)	45 (33–57)	0.003
Noradrenaline (µg/kg/min)	0.5 (0.3–0.6)	0.5 (0.4–0.6)	0.4 (0.3–0.5)	0.005
Vasopressin (U/min)	1.2 (0.6–1.8)	1.6 (1–1.8)	1 (0–1.8)	0.003
MAP (mmHg)	83 (73–95)	81 (72–92)	87 (73–97)	0.179
Heart rate (beats/min)	96 (85–106)	101 (88–110)	93 (82–100)	0.012
CVP (mmHg) ^a^	11 (8–14)	12 (8–14)	11 (8–14)	0.647
VIS	54 (38–66)	56 (46–71)	45 (33–57)	0.003
Body temperature (°C)	36.8 (36.4–37.4)	36.9 (36.4–37.7)	36.8 (36.4–37.0)	0.220
Lactate level (mmol/L)	2.9 (1.9–4.8)	3.5 (2.0–5.7)	2.2 (1.6–3.5)	0.0027
PaO_2_/F_I_O_2_ ratio (mmHg)	256 (163–340)	260 (148–347)	245 (165–337)	0.922
Interventions during ICU stay				
Mechanical ventilation, n (%)	120 (98)	65 (97)	55 (100)	0.501
Continuous renal replacement therapy, n (%)	121 (99)	67 (100)	54 (98)	0.451
Low-dose hydrocortisone, n (%)	106 (87)	63 (94)	43 (78)	0.014
Recombinant soluble thrombomodulin, n (%)	95 (78)	58 (87)	37 (67)	0.015
Antithrombin, n (%)	116 (95)	65 (97)	51 (93)	0.408
Outcomes				
28-day mortality, (%)	17 (14)	13 (19)	4 (7)	0.068
In-hospital mortality, (%)	31 (25)	20 (30)	11 (20)	0.296

Data are presented as median (interquartile range) or count (%).

*PP* peripheral perfusion*, PAI* pulse-amplitude index, *APACHE* acute physiology and chronic health evaluation, *SOFA* sequential organ failure assessment, *JAAM* Japanese Association for Acute Medicine, *DIC* disseminated intravascular coagulation, *PMX-DHP* polymyxin B-direct hemoperfusion, *MAP* mean arterial pressure, *CVP* central venous pressure, *VIS* vasoactive inotropic score, *ICU* intensive care unit.

^a^ CVP values were missing in eight cases (four cases each in the abnormal and normal PP groups).

The implementation of PMX-DHP was as follows: the time from ICU admission to PMX-DHP initiation was significantly shorter (1.7 [0.8–2.6] h vs. 2.4 [1.0–4.2] h, *p* = 0.027) in the abnormal PP group. Two sessions of PMX-DHP were performed in 69% and 62% of the patients in the abnormal and normal PP groups, respectively. The remaining patients completed only one session based on the attending intensivist’s judgment that noradrenaline and vasopressin could be reduced or discontinued and that their hemodynamics were markedly improved. The total duration of PMX-DHP was slightly longer in the abnormal PP group, but the difference was not significant (28.0 [22.7–45.9] h vs. 24.1 [16.8–35.9] h, *p* = 0.097).

### Changes in PAI, VIS, MAP, and other parameters after the initiation of PMX-DHP

**Figure 2:**
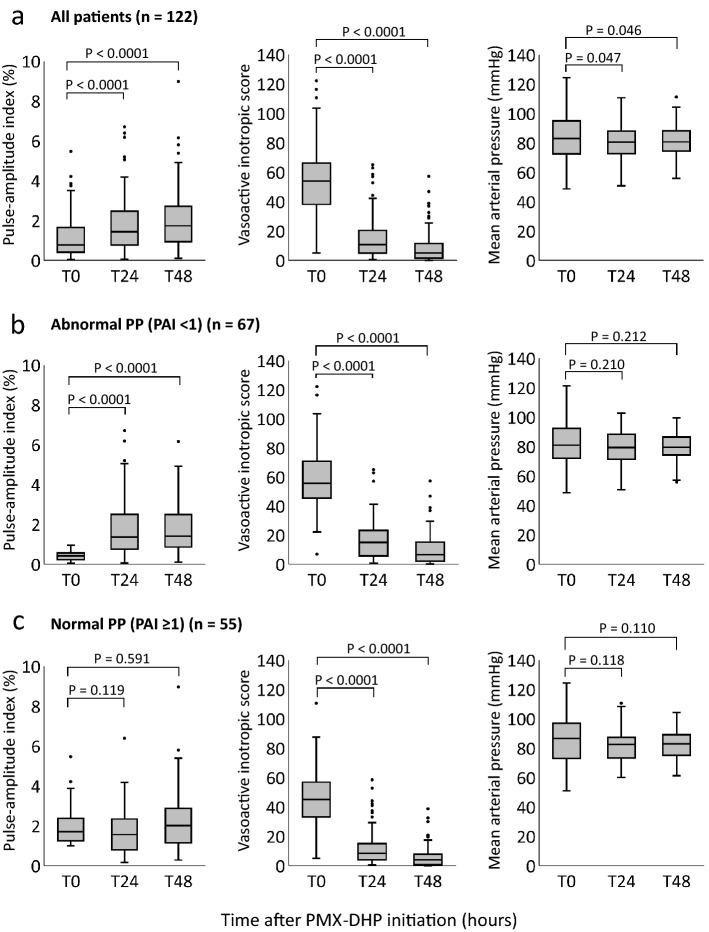
Trends in the pulse-amplitude index, vasoactive inotropic score, and mean arterial pressure at initiation of PMX-DHP (T0) and after 24 (T24) and 48 (T48) h. (**a**) Shows the values of each parameter in all patients, (**b**) shows the values for the abnormal PP group, and (**c**) shows the values for the normal PP group. *PP* peripheral perfusion, *PMX-DHP* polymyxin B-direct hemoperfusion.

Changes in PAI and other parameters after the initiation of PMX-DHP are shown in Fig. 2 and Table 2. In the analysis of all patients, the PAI significantly increased at both T24 (1.44 [0.77–2.47] %, *p* < 0.0001) and T48 (1.74 [0.95–2.71] %, *p* < 0.0001) compared with that at T0 (0.78 [0.41–1.65] %). Conversely, VIS, one of the secondary outcomes, significantly decreased at both T24 (11 [5–19], *p* < 0.0001) and T48 (5 [2–10], *p* < 0.0001) compared with that at T0 (54 [38–66]), while the MAP was maintained (Fig. 2a). The results of subgroup analysis showed that the marked improvement in PAI in the abnormal PP group contributed to the improvement in PAI in the overall patient analysis (Fig. 2b, c). Both subgroups had significantly lower VIS after starting PMX-DHP, but there was no significant change in PAI in the normal PP group during the study period (Fig. 2b, c).

**Table 2:** Changes in heart rate, CVP, and other parameters after the initiation of PMX-DHP.

	T0	T24	T48	*p-*value T0 versus T24	*p*-value T0 versus T48
Heart rate (beats/min)					
All patients (n = 122)	96 (85–106)	88 (79–100)	86 (75–98)	< 0.0001	< 0.0001
Abnormal PP (PAI < 1) (n = 67)	101 (88–110)	87 (79–100)	87 (76–97)	< 0.0001	< 0.0001
Normal PP (PAI ≥ 1) (n = 55)	93 (82–100)	88 (79–104)	86 (72–98)	0.481	0.039
CVP (mmHg) ^a^					
All patients (n = 122)	11 (8–14)	10 (8–12)	9 (7–11)	0.008	< 0.0001
Abnormal PP (PAI < 1) (n = 67)	12 (8–14)	10 (8–13)	9 (7–12)	0.283	0.006
Normal PP (PAI ≥ 1) (n = 55)	11 (8–14)	9 (7–12)	8 (6–11)	0.006	< 0.0001
Body temperature (°C)					
All patients (n = 122)	36.8 (36.4–37.4)	36.6 (36.3–36.9)	36.6 (36.3–37.1)	0.015	0.047
Abnormal PP (PAI < 1) (n = 67)	36.9 (36.4–37.7)	36.5 (36.2–36.9)	36.6 (36.1–36.9)	0.024	0.003
Normal PP (PAI ≥ 1) (n = 55)	36.8 (36.4–37.0)	36.6 (36.3–37.0)	36.7 (36.4–37.2)	0.301	0.719
Lactate level (mmol/L)					
All patients (n = 122)	2.9 (1.9–4.8)	2.0 (1.3–2.8)	1.4 (1.0–2.1)	< 0.0001	< 0.0001
Abnormal PP (PAI < 1) (n = 67)	3.5 (2.0–5.7)	2.1 (1.4–2.9)	1.6 (1.2–2.1)	< 0.0001	< 0.0001
Normal PP (PAI ≥ 1) (n = 55)	2.2 (1.6–3.5)	1.7 (1.2–2.5)	1.2 (0.9–2.1)	< 0.0001	< 0.0001
PaO_2_/F_I_O_2_ ratio (mmHg)					
All patients (n = 122)	256 (163–340)	277 (198–326)	287 (214–349)	0.961	0.079
Abnormal PP (PAI < 1) (n = 67)	260 (148–347)	265 (184–326)	268 (210–361)	0.586	0.285
Normal PP (PAI ≥ 1) (n = 55)	245 (165–337)	286 (213–326)	301 (231–340)	0.505	0.145

Data are presented as median (interquartile range).

*CVP* central venous pressure, *PMX-DHP* Polymyxin B-direct hemoperfusion, *PP* peripheral perfusion, *PAI* pulse-amplitude index.

^a^ CVP values were missing at all measurement points in eight cases (four cases each in the abnormal and normal PP groups).

The heart rates, CVP, body temperatures, lactate levels, and PaO_2_/F_I_O_2_ ratios are shown in Table 2. The heart rates, CVP, and lactate levels were significantly lower at T24 and T48 than at T0 in both groups, but no significant change was observed in the PaO_2_/F_I_O_2_ ratios. Additionally, body temperature was significantly lower at T24 and T48 than at T0 in the abnormal PP group. In contrast, there were no significant differences in body temperature in the normal PP group throughout the study period.

### Correlation between PAI and VIS at PMX-DHP initiation

Overall, PAI was very weakly and negatively correlated with VIS (Spearman’s ρ = − 0.257; *p* = 0.004).

### Cumulative fluid balance after the initiation of PMX-DHP

Table 3 shows the cumulative fluid balance at T24 and T48 after the initiation of PMX-DHP for all patients and in the two subgroups. The 24-h cumulative fluid balance per body weight in the abnormal PP group was significantly higher than that in the normal PP group (80.0 [35.4–113.7] mL/kg vs. 23.1 [3.2–78.5] mL/kg, *p* < 0.0002), but there was no significant between-group difference in the 48-h cumulative fluid balance per body weight (91.3 [27.8–149.8] mL/kg vs. 46.3 [− 6.4 to 105.2] mL/kg, *p* = 0.064).

**Table 3:** Cumulative fluid volume 24 (T24) and 48 (T48) h after initiation of PMX-DHP.

	All patients (n = 122)	Abnormal PP (PAI < 1) (n = 67)	Normal PP (PAI ≥ 1) (n = 55)	*p*-value
24-h cumulative fluid balance (mL)	2978 (637 to 5141)	4248 (1893 to 5755)	1329 (187 to 3704)	0.0005
24-h cumulative fluid balance / BW (mL/kg)	59.2 (9.7 to 99.5)	80.0 (35.4 to 113.7)	23.1 (3.2 to 78.5)	0.0002
48-h cumulative fluid balance (mL)	4348 (560 to 6575)	5081 (1807 to 7190)	2417 (− 478 to 5508)	0.053
48-h cumulative fluid balance / BW (mL/kg)	75.4 (11.1 to 124.8)	91.3 (27.8 to 149.8)	46.3 (− 6.4 to 105.2)	0.064

Data are presented as median (interquartile range) or count (%).

*PMX-DHP* Polymyxin B-direct hemoperfusion, *PP* peripheral perfusion, *PAI* pulse-amplitude index, *BW* body weight.

The results of simple and multiple linear regression analyses of 24-h cumulative fluid balance per body weight and PAI at T0, age, APACHE II score, VIS, immunosuppression status, and abdominal sepsis are summarized in Table 4. PAI, along with abdominal sepsis, was an independent factor associated with the 24-h cumulative fluid balance per body weight (coefficient, − 15.8 [95% confidence interval (CI) − 25.9 to − 5.7]; *p* = 0.0024).

**Table 4:** Simple linear regression and multivariable linear regression analysis of cumulative fluid balance per body weight 24 h after PMX-DHP initiation and PAI at T0, age, APACHE II score, VIS at T0, immunosuppression status, and abdominal sepsis.

Variables	Univariate			Multivariable		
	Coefficient	95% CI	*p-*value	Coefficient	95% CI	*p*-value
Age (years)	0.5	− 0.5 to 1.4	0.330	− 0.1	− 1.0 to 0.9	0.898
APACHE II score	1.1	− 0.4 to 2.6	0.162	0.8	− 0.7 to 2.3	0.276
VIS	0.2	− 0.3 to 0.7	0.414	0.1	− 0.4 to 0.6	0.655
Immunosuppression	3.5	− 26.7 to 33.7	0.818	2.6	− 26.7 to 31.8	0.862
Abdominal sepsis	32.6	11.1 to 54.0	0.0032	31.9	10.2 to 53.5	0.0043
PAI (%)	− 18.9	− 28.6 to − 9.3	0.0002	− 15.8	− 25.9 to − 5.7	0.0024

Data are presented as median (interquartile range) or count (%).

*PMX-DHP* polymyxin B-direct hemoperfusion, *PAI* pulse-amplitude index, *APACHE* acute physiology and chronic health evaluation, *CI* confidential interval, *VIS* vasoactive inotropic score.

## Discussion

In this retrospective exploratory study, PP significantly improved in patients with vasopressor-dependent septic shock at 24 and 48 h along with a significant decrease in vasopressor dose. The results were largely due to a significant improvement in PP in the abnormal PP group in the subgroup analysis. In patients with normal PP, vasopressor doses were reduced without compromising PP. However, fluid balance was significantly higher in patients with abnormal PP.

Recently, many studies on PP and its prognosis have reported that abnormal PP is associated with a worse prognosis not only in patients with septic shock^4–7^ but also in critically ill patients such as those with cardiogenic shock^37^ and those undergoing high-risk surgery^38^. Improving abnormal PP may increase survival rates in patients with septic shock^8,9^, but no specific intervention has yet been established. In a study of patients with cardiogenic shock, macrocirculation, including cardiac index, blood pressure, and heart rate, did not correlate with PP^37^. A similar situation can be inferred for patients with septic shock^39^ wherein it will be difficult to improve abnormal PP with interventions aimed at improving macrocirculation alone, such as inotropic agents and fluid therapy. Indeed, dobutamine administration improves macrocirculation but not PP (as assessed using CRT)^10^.

Conversely, the vasopressors noradrenaline and vasopressin are considered the first- and second-line drugs for patients with septic shock, respectively, as they correct vascular tone depression and improve organ perfusion pressure^17^. However, they also cause peripheral (digital, limb, and mesenteric) ischemia because of PP impairment induced by their vasoconstrictive effects^17,18^. Therefore, interventions aimed at reducing or discontinuing these drugs may effectively improve PP especially in cases with abnormal PP, also called cold shock. This is because administering vasopressors in a state of abnormal PP is not physiologically and pharmacologically rational and may cause adverse effects in patients^40^.

In this study, the dose of vasopressors in the abnormal PP group was significantly reduced 24 and 48 h after the initiation of PMX-DHP, while the PP improved. A negative correlation has been reported between vasopressor dose and PP^41^, and our results showed a similar correlation, albeit feeble. Therefore, we assume that the reduction in vasopressor dose may have been a factor that effectively improved abnormal PP, and PMX-DHP may be a useful intervention for patients with cold shock requiring vasopressors. A preliminary RCT of 28 patients with septic shock also reported that PMX-DHP significantly improved sublingual microcirculation along with a decrease in the noradrenaline dose^42^. Meanwhile, in the normal PP group, VIS significantly decreased without worsening PP. Using vasopressors in vasodilatory shock, also called warm shock, is rational. However, although PP has not been evaluated, a high VIS was reported as an independent prognostic risk factor in patients with septic shock^43^. Thus, reducing VIS may be critical for improving the prognosis of these patients.

After the initiation of PMX-DHP, the hemodynamics of patients in the abnormal and normal PP groups improved and stabilized. Meanwhile, the cumulative fluid balance significantly increased in the abnormal PP group at 24 h. Additionally, PAI at PMX-DHP initiation was an independent factor associated with cumulative fluid balance per body weight in the next 24 h. This result was consistent with a previous study showing a correlation between microcirculatory failure and cumulative fluid balance^20^. Two mechanisms may be responsible for this. First, in patients with shock, blood shifts from peripheral tissues, such as the skin and gastrointestinal tract, to more vital organs (brain, heart, lungs, and kidneys) and skeletal muscles; this is called the “fight or flight response”^44^. Thus, during recovery from peripheral hypoperfusion, an increase in fluid requirement may occur because of blood redistribution. Indeed, fluid loading has been suggested as one of the basic managements for hypotension that occurs when decreasing the noradrenaline dose^45^. Second, when abnormal PP with prolonged CRT and mottled skin is present, hypoperfusion of the gastrointestinal tract occurs simultaneously^46,47^, which may contribute to increased fluid requirements because of concurrent gastrointestinal ischemia and secondary sepsis. Enterocyte injury due to hypoperfusion caused by septic shock has been reported to be significantly associated with intestinal ischemia^48,49^ and increased cumulative fluid balance^50^. A positive fluid balance early after admission to the ICU is associated with worse survival^36,51^. However, it is unclear whether this is because of iatrogenic fluid overload or patient-specific characteristics^50^. We believe that there is a rationale for the mechanism of positive fluid balance during recovery from abnormal PP, and that uniform fluid restriction may worsen PP.

To our knowledge, this is the first report of modifications in PP, hemodynamics, and fluid balance after the initiation of PMX-DHP in patients with vasopressor-dependent septic shock. Abnormal PP, considered a poor prognostic factor, improved significantly after starting PMX-DHP, suggesting that PMX-DHP may be an effective treatment especially for septic patients with abnormal PP. However, caution should be exercised because fluid requirements may differ depending on the PP status.

This study has some limitations. First, this study was a retrospective exploratory analysis. Therefore, our findings need to be confirmed by an RCT with a control group. Additionally, the effect of PMX-DHP on prognosis and organ damage, especially in patients with abnormal PP, needs to be further analyzed. Second, PAI derived from pulse oximetry was used as a surrogate parameter of PP in this study. Currently, neither PAI nor perfusion index has been fully established as an index of PP, and the cutoff value for peripheral hypoperfusion and the normal range has not yet been determined. However, these are promising indices in principle, and future studies are expected. Third, PMX-DHP improves the cardiac index^52^, and septic cardiomyopathy is considered a cause of abnormal PP^53^. Cardiac function was not formally evaluated and recorded in this study; thus, we cannot discuss its association with PP. It is possible that the improvement in cardiac function with PMX-DHP contributed to the improvement in peripheral perfusion, which needs to be clarified in future studies.

Fourth, factors other than PMX-DHP may have influenced the improvement in abnormal PP. This study used low-dose hydrocortisone in approximately 90% of the cases. Hydrocortisone contributes to faster recovery from shock^1,2^ and reduced use of vasopressors. Therefore, it is possible that hydrocortisone positively impacted the present results. Additionally, abnormal PP, such as that resulting in mottled skin and cold shock, is associated with vascular endothelial injury^53,54^ whose causes include DIC^55^. This study used recombinant soluble thrombomodulin and antithrombin for DIC treatment in most cases. These anticoagulants have potential vascular endothelial protective effects^56,57^, which may also have positively influenced our results. Furthermore, previous PMX-DHP RCTs have reported failure to complete planned treatment because of circuit clotting^58^. Therefore, it is possible that these anticoagulants, in addition to nafamostat mesylate, allowed PMX-DHP treatment to be prolonged, thus positively influencing the results. Finally, endotoxin levels were not measured in this study. In this study, gram-negative bacteria were involved in approximately 40% of the cases, and it is unclear to what extent endotoxin adsorption, which is the primary function of PMX-DHP, affected the results of this study. Therefore, future studies are expected to elucidate the exact mechanisms that mediate our promising results on PP.

## Conclusion

Our retrospective exploratory showed that abnormal PP in patients with vasopressor-dependent septic shock significantly improved after the initiation of PMX-DHP. The positive effect may be partly because of the reduction in the vasopressor dose due to the blood pressure-raising effect of PMX-DHP. PMX-DHP may be an effective intervention to improve abnormal PP; however, caution should be exercised as fluid requirements may in patients with abnormal PP may differ from that in patients with normal PP. These findings, including the exact mechanisms of action and prognosis and organ damage-improving effects, must be confirmed by an RCT with a PMX-DHP non-treatment control group.

## Supplementary Information

Supplementary Table S1.

## Data Availability

The datasets used and analyzed during the current study are available from the corresponding author on reasonable request.
