# Supplementary material for: Modifications of peripheral perfusion in patients with vasopressor-dependent septic shock treated with polymyxin B-direct hemoperfusion
Source: Sci Rep. 2023 May 5;13:7295. doi: 10.1038/s41598-023-34084-0 (PMC10163011; doi:10.1038/s41598-023-34084-0)
Supplement: Supplementary file 1 — Supplementary Table S1. [file 41598_2023_34084_MOESM1_ESM.docx]

**Modifications of peripheral perfusion in patients with vasopressor-dependent septic shock treated with polymyxin B-direct hemoperfusion**

Motohiro Sekino^1*⸸^, Yu Murakami^2⸸^, Shuntaro Sato^3^, Ryosuke Shintani^1^, Shohei Kaneko^1^, Naoya Iwasaki^1^, Hiroshi Araki^1^, Taiga Ichinomiya^1^, Ushio Higashijima^1^, Tetsuya Hara^1^

Additional File 1. Baseline characteristics of patients

|  | **All patients (n = 122)** | **Abnormal PP (PAI <1) (n = 67)** | | **Normal PP (PAI ≥1) (n = 55)** | | *p*-value |
| --- | --- | --- | --- | --- | --- | --- |
| Admission route to the ICU |  |  |  | |  | |
| Emergency department or other hospitals, n (%) | 102 (84) | 55 (82) | 47 (85) | | 0.617 | |
| Hospital ward, n (%) | 20 (16) | 12 (18) | 8 (15) | | 0.807 | |
| Surgical intervention before ICU admission, n (%) | 79 (65) | 44 (66) | 35 (64) | | 0.851 | |
| Comorbidity |  |  |  | |  | |
| Hypertension, n (%) | 56 (46) | 26 (39) | 30 (55) | | 0.101 | |
| Chronic heart failure, n (%) | 45 (37) | 25 (37) | 20 (36) | | 1.000 | |
| Diabetes mellitus, n (%) | 38 (31) | 17 (25) | 21 (38) | | 0.169 | |
| Chronic kidney disorders, n (%) | 33 (27) | 18 (27) | 15 (27) | | 1.000 | |
| Liver failure, n (%) | 19 (16) | 11 (16) | 8 (15) | | 0.808 | |
| Chronic obstructive pulmonary disease, n (%) | 15 (12) | 9 (13) | 6 (11) | | 0.785 | |
| Immunosuppression, n (%) | 19 (16) | 10 (15) | 9 (16) | | 1.000 | |
| Cancer or hematological malignancy |  |  |  | |  | |
| Remission, n (%) | 17 (14) | 9 (13) | 8 (15) | | 1.000 | |
| Evolutive, n (%) | 15 (12) | 9 (13) | 6 (11) | | 0.785 | |
| Site of infection |  |  |  | |  | |
| Abdomen, n (%) | 71 (58) | 41 (61) | 30 (55) | | 0.468 | |
| Lung/thorax, n (%) | 22 (18) | 10 (15) | 12 (22) | | 0.352 | |
| Urinary tract, n (%) | 8 (7) | 6 (9) | 2 (3.6) | | 0.292 | |
| Soft tissue, n (%) | 9 (7) | 6 (9) | 3 (5.5) | | 0.511 | |
| Other/unknown, n (%) | 12 (10) | 4 (6) | 8 (15) | | 0.135 | |
| Causative microorganism |  |  |  | |  | |
| Gram-positive, n (%) | 33 (27) | 22 (33) | 11 (20) | | 0.151 | |
| Gram-negative, n (%) | 27 (22) | 14 (21) | 13 (24) | | 0.827 | |
| Mixed, n (%) | 25 (20) | 13 (19) | 12 (22) | | 0.823 | |
| Mixed with gram-negative | 20 (16) | 9 (13) | 11 (20) | | 0.340 | |
| Gram-negative + mixed with gram-negative | 47 (39) | 23 (34) | 24 (44) | | 0.351 | |
| Fungi, n (%) | 6 (5) | 2 (3) | 4 (7) | | 0.408 | |
| Unknown, n (%) | 31 (25) | 16 (24) | 15 (27) | | 0.682 | |
| Bacteremia, n (%) | 48 (39) | 29 (43) | 19 (35) | | 0.356 | |

Data are presented as count (%)

*PP* peripheral perfusion, *PAI* pulse-amplitude index, *ICU* intensive care unit
